# Genomewide selection for fruit quality traits in apple: breeding insights gained from prediction and postdiction

**DOI:** 10.1093/hr/uhad088

**Published:** 2023-05-03

**Authors:** Sarah A Kostick, Rex Bernardo, James J Luby

**Affiliations:** Department of Horticultural Science, University of Minnesota, Saint Paul, MN 55108, USA; Department of Agronomy and Plant Genetics, University of Minnesota, Saint Paul, MN 55108, USA; Department of Horticultural Science, University of Minnesota, Saint Paul, MN 55108, USA

## Abstract

Many fruit quality traits in apple (*Malus domestica* Borkh.) are controlled by multiple small-effect quantitative trait loci (QTLs). Genomewide selection (genomic selection) might be an effective breeding approach for highly quantitative traits in woody perennial crops with long generation times like apple. The goal of this study was to determine if genomewide prediction is an effective breeding approach for fruit quality traits in an apple scion breeding program. Representative apple scion breeding germplasm (n_individuals_ = 955), high-quality single nucleotide polymorphism (SNP) data (n_SNPs_ = 977), and breeding program fruit quality trait data at harvest were analyzed. Breeding parents `Honeycrisp' and `Minneiska' were highly represented. Moderate to high predictive abilities were observed for most fruit quality traits at harvest. For example, when 25% random subsets of the germplasm set were used as training sets, mean predictive abilities ranged from 0.35 to 0.54 across traits. Trait, training and test sets, family size for within family prediction, and number of SNPs per chromosome affected model predictive ability. Inclusion of large-effect QTLs as fixed effects resulted in higher predictive abilities for some traits (e.g. percent red overcolor). Postdiction (i.e. retrospective) analyses demonstrated the impact of culling threshold on selection decisions. The results of this study demonstrate that genomewide selection is a useful breeding approach for certain fruit quality traits in apple.

## Introduction

Apple (*Malus domestica* Borkh.) breeding is a resource-intensive, time-consuming process; therefore, apple breeders are always looking for ways to make the breeding pipeline more efficient. Significant emphasis has been placed on identification and characterization of quantitative trait loci (QTLs) underlying breeding relevant traits. Although many QTLs associated with fruit appearance and quality traits in apple have been reported (summarized by Teh et al. [1]), few have been translated into locus-specific trait-predictive DNA tests for use in breeding (discussed in Teh et al. [1] and Evans and Peace [2]). In addition, many apple fruit quality traits are genetically complex as they are likely controlled by many small-effect QTLs throughout the genome. For example, QTLs throughout the genome including chromosomes (Chrs) 1, 2, 3, 8, 12, 13, 15, and 16 have been reported for soluble solids content (SSC) [1, 3–5]. Finally, apple is characterized by a long juvenility period and phenotyping fruit quality traits is challenging. Genomewide selection (genomic selection) might be a valuable breeding approach when targeting highly quantitative traits in apple.

Genomewide selection, which was described in the seminal work by Meuwissen et al. [6], aims to predict performance of an individual using hundreds to thousands of randomly distributed markers. Predictive ability of genomewide models, the correlation (*r*) between the observed and predicted values of test set individuals [7], has been examined extensively in plant systems with the primary focus being on crops such as barley (*Hordeum vulgare* L.), maize (*Zea mays* L.), soybean (*Glycine max* (L.) Merr.), and wheat (*Triticum aestivum* L.) (reviewed by Krishnappa et al. [8]). In apple, several studies have focused on estimating predictive abilities of models for various breeding-relevant traits including percent red overcolor, fruit weight, russet formation, multiple fruit texture traits, SSC, titratable acidity (TA), and multiple polyphenols [9–19]. Factors such as trait heritability, the number of QTLs underlying the trait (genetic architecture), size of training set, relatedness between training and test sets, marker density, and statistical model applied can affect predictive ability (reviewed by Krishnappa et al. [8]). Therefore, predictive abilities for a given trait have often varied widely from study-to-study.

The University of Minnesota (UMN) apple breeding program scheme has three main phases of evaluation: 1) unreplicated seedling testing; 2) clonal testing; and 3) grower trials/pre-commercialization testing [20]. In each phase of the breeding program, the number of unique individuals decreases and the number of replicates (i.e., clones) per individual increases in comparison to the previous evaluation phase. In the first phase of the apple breeding program (i.e. unreplicated seedlings), fruit from individual seedling trees are sampled by breeding program personnel in the orchard at an appropriate maturity stage and culled or selected for evaluation in clonal testing based on the overall eating experience at harvest. Fruit availability is limited and therefore, extensive phenotyping procedures are not utilized during the unreplicated seedling phase. Genomewide selection might be a useful approach to identify potential advanced selections in the first phase of the breeding program.

Few studies have examined the utility of genomewide prediction in an apple breeding program. In this study, our goal was to determine the utility of genomewide prediction within the context of the UMN apple breeding program for several fruit quality traits: percent red overcolor, fruit weight, two instrumental firmness measures (M1, M2), an instrumental crispness estimate (Cn), SSC, and TA. Prediction and postdiction (retrospective explanation), a term that has been used in previous studies [e.g., 21], were used to determine the potential usefulness of genomewide prediction. Representative `Honeycrisp'-derived breeding germplasm, high-quality single nucleotide polymorphism (SNP) data, and breeding program fruit quality trait data at harvest were analyzed to test the hypotheses that: 1) use of genomewide prediction would result in moderate to high predictive abilities for certain fruit quality traits; 2) high heritability traits (e.g., percent red overcolor), larger training sets, and higher marker densities would result in higher predictive abilities; 3) genomewide selection would have performed similarly to phenotypic selection; and 4) advanced selections would have been identified via genomewide selection.

## Results

### Trait variation

**Figure 1:**
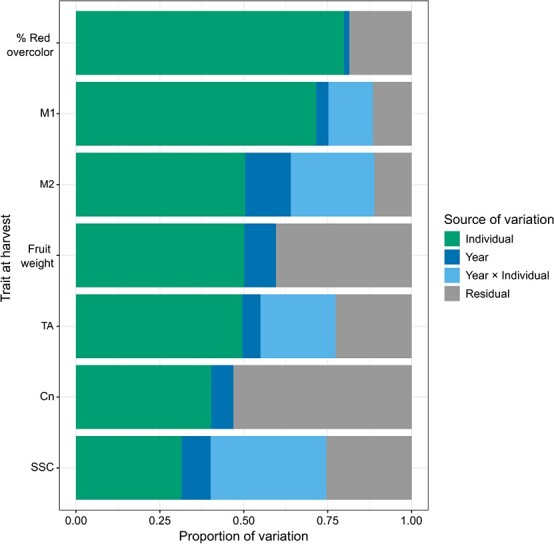
Proportion of phenotypic variation associated with individual (i.e., advanced selection, cultivar, parent, progenitor, or unselected offspring), year, year × individual interaction (if applicable), and residual error effects for percent red overcolor, fruit weight, instrumental texture traits (M1, M2, Cn), soluble solids content (SSC), and titratable acidity (TA). Year × interaction term was not included in percent red overcolor, fruit weight, and Cn models.

**Figure 2:**
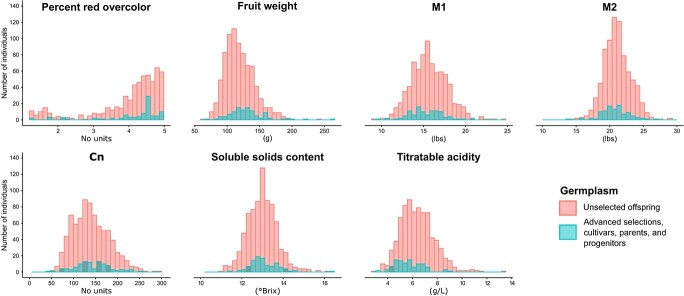
Distributions of across-year adjusted trait best unbiased predictions (BLUPs) for phenotyped unselected offspring and other related breeding germplasm (i.e., advanced selections, cultivars, parents, and progenitors). Germplasm described in Table 2.

Year (*p* < 0.001) and individual (*p* < 0.0001) had significant effects on all traits. Normal QQ plots (i.e. sample vs. theoretical quantiles plots) indicated that random effects were normally distributed for most traits (Figure S1). Across traits, the mean proportion of variation accounted for by year was 0.07 and the mean proportion of variation accounted for by individual was 0.53. For all traits except M2, year accounted for ≤0.10 of trait variation (Figure 1). Individual accounted for ≥0.50 of trait variation for percent red overcolor, fruit weight, M1, M2, and TA whereas individual accounted for ≤0.40 of trait variation for Cn and SSC (Figure 1). For M1, M2, SSC, and TA, a year × individual interaction term was included in the model and accounted for 0.13 (M1) to 0.34 (SSC) of the variation (Figure 1). Quantitative variation for most traits, quantified as across year trait best linear unbiased predictions (referred to throughout as trait BLUPs), was observed among individuals in the germplasm set (Figure 2). The distribution of percent red overcolor BLUPs was skewed toward high ratings (4.0 to 5.0; Figure 2).

### Prediction across germplasm set

**Figure 3:**
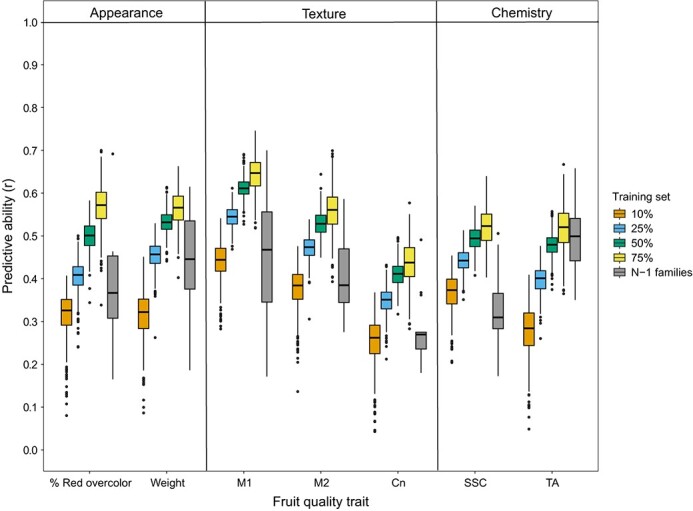
Genomewide prediction model predictive abilities (*r*) for percent red overcolor, fruit weight, fruit flesh texture traits (M1, M2, Cn), soluble solids content (SSC) and titratable acidity (TA) best linear unbiased predictions (BLUPs) at harvest. All SNPs (n = 977) were included as random effects. Five different training sets were used: 10, 25, 50, and 75% random samples of entire population used as training set (500 iterations) and all but one family (N – 1) as training set (each family used as test set once).

Moderate to high predictive abilities were observed for most fruit quality traits at harvest (Figure 3; Table S1). Across the seven fruit quality traits, mean predictive abilities ranged from 0.25 to 0.44 when 10% of population was used as training set, from 0.35 to 0.54 when 25% of population used as training set, from 0.41 to 0.61 when 50% of population was used as training set, and from 0.44 to 0.64 when 75% of population was used as training set (Figure 3; Table S1). Regardless of the trait, the larger the training set, the higher the predictive ability (Figure 3; Table S1). Variation for predictive ability was observed across iterations for a given training set size (Figure 3). Predictive abilities were greater than zero across all iterations and traits (Table S1). When an untested family was used as the test set, predictive abilities ranged from 0.28 to 0.50 for the seven traits (Figure 3; Table S1). More variation for predictive ability was observed when untested families were used as test sets compared to when random subsets of the whole population were used (Figure 3). In general, the highest predictive abilities were observed for M1 (mean = 0.56) while the lowest predictive abilities were observed for Cn (mean = 0.36; Figure 3; Table S1).

### SNPs per chromosome and predictive ability

Inclusion of more SNPs per chromosome was generally associated with higher predictive abilities when an individual family was used as the test set (Figure S2). Across test families and traits, mean predictive abilities ranged from 0.03 to 0.44 for five SNPs sampled per chromosome, 0.08 to 0.58 for 10 SNPs per chromosome, 0.11 to 0.64 for 15 SNPs per chromosome, 0.13 to 0.66 for 20 SNPs per chromosome, 0.11 to 0.68 for 25 SNPs per chromosome, and 0.14 to 0.70 for 30 SNPs per chromosome. The number of SNPs per chromosome, trait, and test family had significant effects on predictive ability (Figure S2). The largest differences in mean predictive ability were observed between five and 10 SNPs sampled per chromosome with mean predictive abilities being 0.06 to 0.09 greater when 10 SNPs were sampled versus five SNPs (Figure S2). Significant variation in predictive abilities was observed across iterations regardless of the number of SNPs sampled (Figure S2).

### Prediction within families

When individual offspring within families were used as the test set, predictive abilities within families varied significantly by family and trait (Table 1). Mean predictive abilities ranged from −0.20 to 0.26 across traits and families (Table 1). Pearson correlation coefficients between family size (number of phenotyped offspring) and within family predictive ability ranged from 0.33 to 0.60 across traits with significant correlations for fruit weight, Cn, and SSC (Table 1). In general, families with more phenotyped offspring had higher predictive abilities compared to families with fewer phenotyped offspring regardless of the trait. Mean family sizes of families with predictive abilities greater than zero ranged from 66 to 89 phenotyped offspring across traits. In contrast, mean family sizes of families with negative predictive abilities ranged from 33 to 43 phenotyped offspring across traits. For most traits, except for SSC and TA, positive correlation coefficients were observed for predictive ability and within family trait variation, quantified as trait standard deviation (Table 1). However, predictive ability and within family trait variation were only significantly correlated for Cn (Table 1).

**Table 1:** Summary of within family genomewide prediction for trait best linear unbiased predictions (BLUPs) at harvest

Trait at harvest	Family		Predictive ability^c^			Summary of families with predictive abilities						Predictive ability correlations (*r*)^f^	
						**Greater than zero** ^d^			**Less than zero** ^e^				
	**Mean size** ^a^	**Trait SD** ^b^	**Mean**	**Min**	**Max**	**Number**	**Mean family size**	**Mean trait SD**	**Number**	**Mean family size**	**Mean trait SD**	**Family size**	**Trait SD**
% Red overcolor	72	0.85	0.23	−0.08	0.58	6	82	0.85	2	43	0.84	0.51	0.51
Weight (g)	63	21.39	0.03	−1.00	0.64	7	82	22.42	6	41	20.20	0.60*	0.33
M1 (lbs)	61	1.94	0.26	−0.80	0.66	11	66	1.95	2	33	1.90	0.38	0.24
M2 (lbs)	61	1.78	0.12	−0.62	0.51	9	69	1.81	4	43	1.70	0.36	0.22
Cn	61	38.18	−0.20	−1.00	0.47	7	78	40.84	6	42	35.08	0.52·	0.58·
SSC (°Brix)	60	0.60	−0.13	−0.77	0.30	5	89	0.60	8	42	0.60	0.50·	−0.11
TA (g/L)	60	1.33	0.08	−0.78	0.46	8	71	1.33	5	43	1.33	0.33	−0.17

a Mean number of offspring within a family phenotyped for a given trait

b Within family trait variation quantified as within family trait standard deviation (SD)

c Mean, minimum (Min), and maximum (Max) within family predictive abilities for fruit quality traits

d Number of families, mean family size, and mean within family trait standard deviation (SD) for families with within family predictive abilities that were greater than zero

e Number of families, mean family size, and mean within family trait standard deviation (SD) for families with within family predictive abilities that were less than zero

f Pearson correlation coefficients (r) between predictive ability and number of offspring within a family or within family trait standard deviation (SD). Significance designated as follows: ·indicates a p-value <0.10 whereas * indicates a p-value <0.05

Higher predictive abilities were observed with larger family subsets across traits (Figures S3 and S4; Table S2). Large variation in predictive abilities across iterations was observed for small numbers of offspring within the family subsets (Figures S3 and S4). For the `Honeycrisp' × `Minnewashta' family, mean predictive abilities were negative for most traits when ≤40 offspring were included (Figure S3; Table S2). Similar trends were observed for `Minneiska'× “MN55” with mean predictive abilities being negative for most traits when ≤60 offspring were included (Figure S4; Table S2). Similar trends were observed for `Minneiska' × `MN55' with mean predictive abilities being negative for most traits when ≤60 offspring were included (Figure S4; Table S2).

### Inclusion of SNPs at large-effect QTLs as fixed effects

Significant differences in mean trait BLUPs were observed among genotypes of SNPs at Chr9 overcolor locus for percent red overcolor, Chr16 texture QTL for instrumental firmness measurements (M1, M2), and *Ma* + *Ma3* for TA (Table S3). When an untested family was used as the test set, inclusion of SNPs at previously reported large-effect QTLs sometimes resulted in higher predictive abilities depending on the trait and test family (Table S4). When a SNP at Chr9 color locus was included as a fixed effect, predictive abilities for percent red overcolor were 0.02 to 0.48 higher compared to the random effects models (Table S4). For percent red overcolor, the largest increases in model predictive ability were observed when at least one parent of the test family was heterozygous for the fixed-effect SNP (Table S4). For example, predictive ability for percent red overcolor was estimated to be 0.33 for the random effects model and 0.80 for the model that included a SNP at Chr9 color locus as a fixed effect when `Honeycrisp' × 'WA2' offspring were used as the test set (Table S4). Both `Honeycrisp' and `WA2' were heterozygous for the Chr9 SNP (Table S3). Inclusion of a SNP at the Chr16 texture QTL as a fixed effect did not result in significantly different predictive abilities for M1 and M2 (Table S4). Overall, inclusion of SNPs at *Ma3* (Chr8) and *Ma* (Chr16) as fixed effects did not result in significantly higher predictive abilities for TA (Table S4). However, for five test families, inclusion of *Ma3* and *Ma* as fixed effects resulted in an ≥0.10 increase in predictive ability for TA (Table S4).

### Genomewide postdiction

**Figure 4:**
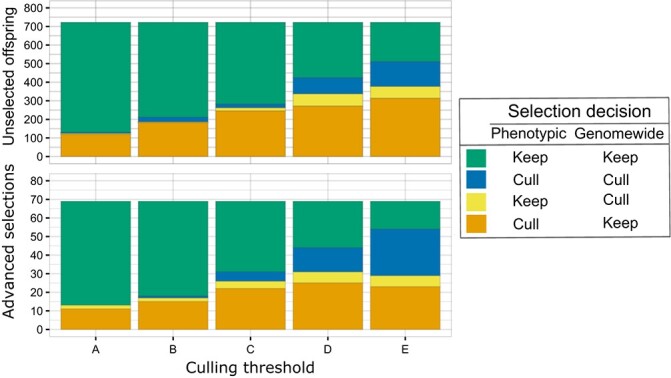
Numbers of unselected offspring (n = 722) and advanced selections (n = 69) that would have been culled or kept using phenotypic selection (phenotypic) and/or genomewide selection (genomewide). Traits included in postdiction analyses were fruit weight, texture traits (M1, M2), soluble solids content (SSC), and titratable acidity (TA). Culling thresholds are described in Tables 3 and S5.

The number of offspring and advanced selections that would have been culled depended on the selection method (i.e., phenotypic selection or genomewide selection) and independent culling thresholds applied (Tables S5 and S6). In general, fewer offspring and advanced selections would have been culled based on their predicted values (genomewide selection) compared to observed trait values (phenotypic selection) (Table S6; Figure 4). Depending on selection thresholds, selection decisions (i.e., keep, cull) for approximately 48 to 82% of offspring and 55 to 81% of advanced selections would have been the same regardless of selection method (i.e., phenotypic selection, genomewide selection; Figure 4).

#### Culling thresholds using `Honeycrisp' trait levels

The fewest number of individuals would have been culled using thresholds estimated with ‘Honeycrisp’s trait values (Threshold A; Tables S5 and S6). Using these culling thresholds, 124 of 722 offspring and 11 of 69 advanced selections would have been culled based on their observed trait values whereas only 11 offspring and two advanced selections would have been culled based on their predicted values (Table S6). Most individuals would have been culled based on only one trait (Table S7) with the highest number of individuals being culled based on observed or predicted M2 values (Table S6). No recently released cultivars (e.g., Minneiska, MN33, MN55, MN80) included in this study would have been culled using either phenotypic or genomewide selection (Table S8).

#### Culling thresholds using mean trait levels of advanced selections in training sets

More individuals would have been culled if culling thresholds estimated using mean trait levels of advanced selections (Threshold B) had been used compared to thresholds estimated with ‘Honeycrisp’s trait values (Tables S5 and S6). Specifically, 207 of 722 offspring and 16 advanced selections including `Minneiska' and `MN55' would have been culled based on observed trait values (Tables S6 and S8). Most individuals would have been culled based on only one trait (Table S7). `Minneiska' would have been culled based on its observed fruit weight BLUP while `MN55' would have been culled based on both its observed fruit weight and SSC BLUPs (Table S8). In contrast to phenotypic selection, 28 unselected offspring and three advanced selections would have been culled based on predicted trait values (Table S6).

#### Culling thresholds using offspring percentile values

High numbers of individuals would have been culled if culling thresholds (Thresholds C, D, E), calculated based on percentile values, had been used (Tables S5 and S6). Using phenotypic selection, 264 to 446 offspring and 27 to 48 advanced selections including `Minneiska', `MN55', and `MN80' would have been culled depending on the selection thresholds used (Tables S6 and S8). Using genomewide selection, 37 to 198 offspring and 31 advanced selections would have been culled depending on the selection thresholds employed. `Minneiska', 'MN33', and `MN55' would have been culled via genomewide selection if thresholds D and E had been used (Tables S6 and S8). Additionally, `MN80' would have been culled using genomewide selection if Threshold E had been applied (Table S8). The highest numbers of individuals would have been culled if thresholds estimated from the 90% percentile for fruit weight and the 25% percentiles for M1, M2, SSC, and TA (Threshold E) had been used (Table S6). The largest disagreements between phenotypic and genomewide selection decisions were observed when applying culling Thresholds D or E (Figure 4).

## Discussion

Results from this study demonstrate that genomewide prediction could be utilized to predict fruit quality traits in apple. Moderate to high predictive abilities were observed for most of the examined fruit quality traits with predictive abilities varying with trait, training-test sets, and SNP number. In postdiction analyses, selection decisions (i.e., keep, cull) for a mean of 64% of unselected offspring and 66% of advanced selections would have been the same regardless of selection method (i.e. phenotypic selection, genomewide selection). Depending on the culling thresholds, 16% to 70% of advanced selections examined would have been culled if phenotypic selection had been used whereas 3% to 45% of advanced selections examined would have been culled if genomewide selection had been used. Results of this study demonstrate the utility as well as challenges of genomewide selection for fruit quality traits in apple breeding programs that utilize `Honeycrisp'-derived germplasm.

### Moderate to high predictive abilities for fruit quality traits across germplasm set

Moderate to high predictive abilities for most fruit quality traits at harvest were observed across training-test sets indicating that genomewide selection is an effective breeding approach for certain traits. Predictive abilities estimated were similar to those reported in previous studies [9–13, 15, 16, 18, 19, 22]. For example, in this study, mean predictive abilities ranged from 0.32 to 0.57 for percent red overcolor (Figure 2) which were similar to the predictive abilities for overcolor (<0.20 to 0.57) reported by McClure et al. [13], Migicovsky et al. [15], Minamikawa et al. [16], Muranty et al. [17], and Zheng et al. [19]. Some studies [e.g., 9, 10] reported higher predictive abilities for percent red overcolor than those observed in this study. Variation in reported predictive abilities for percent red overcolor was likely due to differences in germplasm, trait distribution, experimental design, and models applied.

### Predictive abilities for fruit quality traits varied significantly

Model predictive abilities varied significantly with trait, training set size and composition, and test set. This was not surprising as significant variation for model predictive abilities has been observed within and among previous studies in apple [9–13, 15, 16, 18, 19, 22]. Predictive abilities for breeding relevant traits should be estimated for target breeding germplasm before implementing genomewide selection.

In this study, the proportion of trait variation associated with individual affected predictive abilities. Since many individuals in this study were unreplicated, variation among individuals contained components of both genetic and environmental effects. Previous studies in apple and other crops (e.g. barley, maize, wheat) have demonstrated that high heritability traits often have higher predictive abilities compared to low heritability traits [e.g., 22, 23, 24, 25, 26]. For example, in this study, M1 had the highest mean predictive ability and 72% of M1 phenotypic variation was associated with individual. Traits like M1 could be effectively targeted with genomewide prediction. In contrast, only 40% of the phenotypic variation for Cn, which had the lowest mean predictive ability, was associated with individual. Relatively low proportion of Cn variation associated with individual could partially explain the relatively low predictive abilities associated with Cn*.* As Cn in this study only had moderate predictive abilities, Cn might not be a good target for genomewide prediction and different phenotypic measures of crispness (e.g., sensory evaluation) should be explored. Similar to Cn, a relatively low proportion of the SSC variation was associated with individual (Figure 1). However, SSC is successfully being targeted with genomewide selection in other fruit breeding programs, specifically strawberry (*Fragaria* × *ananassa* Duchesne) [e.g., 27, 28]. Therefore, genomewide selection might be an effective breeding approach for SSC in apple.

### Inclusion of QTLs as fixed effects sometimes resulted in higher predictive abilities

Inclusion of SNPs at previously detected QTLs as fixed effects resulted in higher predictive abilities for certain traits. In this study, including a SNP at the Chr9 red color locus [e.g., 16, 19, 29, 30] as a fixed effect for percent red overcolor was often associated with significantly higher predictive abilities (Table S4). Not surprisingly, the largest increases in predictive ability for percent red overcolor were observed in families expected to segregate at the Chr9 color locus (i.e., at least one parent was heterozygous). For other traits, models that included fixed effects often did not result in significantly higher predictive abilities (Table S4). In a simulation study, Bernardo [31] concluded that large-effect QTLs should be included as fixed effects when only a few genes underlie the trait and when each gene accounts for ≥10% of the genetic variation. In this study, the QTLs included as fixed effects were considered large effect (Table S3); however, it is possible that the QTL effects were not large enough to improve model predictive abilities in some cases (Table S4). Even though inclusion of QTLs as fixed effects did not always result in improved model accuracy, including QTLs as fixed effects was rarely detrimental (Table S4).

### Moderate predictive abilities achieved with relatively few SNPs

Moderate predictive abilities were achieved for most traits using a reduced set of SNPs. In general, more SNPs per chromosome was associated with higher predictive abilities across traits. This was not surprising as previous studies in other crops [e.g., 24] have demonstrated that marker number affects predictive ability with higher marker numbers being associated with higher predictive abilities. Predictive ability began to plateau around 25 SNPs per chromosome (Figure S2), which indicated that marker saturation was potentially reached. For this germplasm set, approximately 500 genomewide SNPs are likely sufficient to achieve moderate to high predictive abilities. The relatively few numbers of SNPs per chromosome required to achieve moderate predictive abilities was likely due to the high relatedness of this germplasm set [32] (Table 2). Most offspring were derived from crosses with `Honeycrisp' or `Minneiska', an offspring of `Honeycrisp', as well as related UMN material (e.g. `Minnewashta', `MN55', MN1702, MN1836, MN1915, MN1965; Table 2). Many offspring in training and test sets likely share large haplotype lengths and therefore, few SNPs were likely needed to represent alleles. It is important to note that SNPs were randomly sampled from the 977 SNPs retained after removal of SNPs that were monomorphic, redundant, or had high percentages of missing data. A limitation of this study was that genetic positions (cM) of sampled SNPs on a given chromosome were not considered. As a result, multiple SNPs with similar genetic positions could have been sampled, which could partially explain the lower model predictive abilities when lower numbers of SNPs were used. Another limitation is that the same number of SNPs per chromosome were sampled regardless of chromosome length. A potentially better approach would be to choose the number of SNPs per chromosome based on chromosome length.

**Table 2:** Summary of apple full-sib family offspring and other germplasm phenotyped for fruit quality traits in at least one year from 2010 to 2020

**Germplasm**	**Family**	**Maternal**	**Paternal**	**Number of phenotyped individuals included** ^**a**^								**Postdiction training set** ^**b**^
				**Total**	**% Red overcolor**	**Weight**	**M1**	**M2**	**Cn**	**SSC**	**TA**	
	1	Dayton	Minnewashta	23	0	23	22	22	22	23	23	1, 2, 3
	2	Honeycrisp	Jonafree	18	0	53	44	44	44	43	43	1, 2, 3
		Jonafree	Honeycrisp	35								
	3	Honeycrisp	MN1702	31	0	52	39	39	39	49	49	1, 2, 3
		MN1702	Honeycrisp	21								
	4	Honeycrisp	MN1836	51	51	49	51	51	51	49	49	1, 2, 3
	5	Honeycrisp	MN1915	48	48	30	48	48	48	38	38	1, 2, 3
	6	Honeycrisp	AA44	75	0	75	50	50	50	75	75	1, 2, 3
Unselected offspring	7	Honeycrisp	Pitmaston Pine Apple	51	0	51	40	40	40	41	40	1, 2, 3
	8	Honeycrisp	WA2	48	46	48	48	48	48	47	47	1, 2, 3
	9	Honeycrisp	Minnewashta	150	145	150	150	150	150	149	149	1, 2, 3
	10	Minneiska	MN1965	50	48	49	50	50	50	43	43	2, 3
	11	Minneiska	MN55	128	120	124	128	128	128	124	124	2, 3
	12	Minneiska	Wildung	82	82	70	82	82	82	61	61	2, 3
	13	MN1702	Minneiska	41	37	41	41	41	41	40	40	2, 3
Advanced selections, cultivars, parents, and progenitors				103	90	103	103	103	103	103	103	Various
Total				955	667	918	896	896	896	885	884	NA

a Numbers of individuals within each family or germplasm type phenotyped for percent red overcolor (% Red overcolor), fruit weight (Weight), instrumental firmness measurements M1 and M2, instrumental crispness estimate Cn, soluble solids content (SSC), and titratable acidity (TA).

b Three rounds of postdiction (retrospective) analyses were carried out to determine if 69 advanced selections selected using traditional selection methods would have been identified via genomewide prediction.

### Within family prediction is a useful breeding approach in large families

Genomewide selection within families is a promising breeding approach (Table 1; Figures S3 and S4). Similar crosses (parental combinations) are often made from year to year. As a result, different age plantings of offspring derived from the same parents are often present in unreplicated seedling orchards. Within-family genomewide prediction models could be developed using fruiting individuals from a given cross. Models could subsequently be used to predict trait levels of related individuals that are 1) established in the orchard but have not yet flowered or 2) seedlings in the greenhouse from a different breeding cycle. To achieve sufficient predictive abilities, relatively large family sizes are needed. Not surprisingly, larger family sizes were generally associated with higher predictive abilities within families in this study (Table 1; Figures S3 and S4). The marginal improvement to predictive ability from adding more individuals decreased substantially when the training set already contained 80 to 100 individuals.

### Insights from genomewide postdiction analyses

#### Traits included in genomewide postdiction analyses

Apple breeders simultaneously target multiple traits with no single trait completely driving selection decisions. For example, the overall consumer experience is created by multiple appearance, texture, and flavor traits and people can vary in their preferences [33]. Therefore, predicting which individuals are likely to provide an excellent overall eating experience can be challenging. Not every trait examined can or should be targeted with genomewide selection. In this study, fruit weight, M1, M2, SSC, and TA were targeted in genomewide postdiction (i.e., retrospective) analyses due to sufficient phenotypic variation (Figure 2), moderate to high predictive abilities (Figure 3), breeding relevance, and/or lack of relevant locus-specific DNA tests for the trait. Percent red overcolor and Cn were not targeted due to relatively low phenotypic variation (Figure 2) or predictive abilities (Figure 3).

#### Phenotypic selection likely more stringent that genomewide selection for individual traits

Culling based on observed values is likely more stringent compared to genomewide selection when considering individual traits. In general, higher numbers of individuals would have been culled using phenotypic selection compared to genomewide selection for individual traits. This was not surprising as most individuals with more extreme phenotypes (i.e., values at the distribution tails) were predicted to have values towards the middle of the distribution. For example, the two offspring with the lowest (9.17 lbs) and highest (24.65 lbs) M1 BLUPs had predicted values of 16.38 and 18.91, respectively, which were similar to the population mean (15.78 lbs). Therefore, as observed by Muranty et al. [17], individuals at the tails of the distributions that normally would be either culled or selected based on their phenotypic values might not be identified with genomewide selection.

During the unreplicated seedling phase of the breeding program, a selection decision for a given individual is made during a single evaluation. Environmental or genotype × environment effects might significantly impact fruit quality traits and therefore the selection decision. In this study, across-year trait BLUPs, which might have accounted for some environmental or genotype × environment effects, were used as phenotypic values. The stringency and accuracy of phenotypic selection based on individual traits or overall eating experience are influenced by the phenotypic data (e.g., across-year trait BLUPs, eating quality in one year) that are relied upon.

#### Determining culling thresholds for fruit quality traits in apple is challenging

In an apple breeding program, individuals are evaluated based on their overall eating experience and stringent culling thresholds for specific fruit quality traits are not often used. Therefore, determining culling thresholds for targeted traits is challenging and could potentially limit the application of genomewide selection. Not surprisingly, the choice of culling thresholds significantly affected the number of individuals that would have been culled or selected for further evaluation regardless of the selection method. Use of trait levels of `Honeycrisp' or advanced selections to set culling thresholds resulted in the culling of few advanced selections. Culling using genomewide selection generally would not have been effective with either of these thresholds as the number of individuals culled would have been very low (≤ 4%). In contrast, some culling thresholds were likely too stringent as released cultivars like `Minneiska', `MN33', and `MN55' would have been culled. For example, 19 advanced selections including `Minneiska', `MN33', and `MN55' would have been culled with genomewide selection using culling thresholds based on the 90% percentile for fruit weight, 10% percentiles for SSC and TA, and the 25% percentiles for M1 and M2 (Tables S6 and S8). A limitation of this study was that all traits were considered independently and to be of relatively equal breeding importance. To inform breeding targets and culling thresholds and enable adoption of genomewide selection, future research should focus on determining what traits and associated trait levels are indicative of high eating quality. Additionally, methods of multi-trait selection such as selection based on a multiple trait index should be explored in apple.

**Table 3:** Breeding goals and independent culling threshold scenarios for postdiction analyses

**Trait**	**Breeding goal**	**Direction** ^**a**^	**Culling threshold scenario** ^**b**^				
			**A**	**B**	**C**	**D**	**E**
% Red overcolor	Various			Keep all			
Fruit weight (g)	Avoid excessively large fruit	≥	Honeycrisp + SD	Selection mean + SD	90%	90%	90%
M1 (lbs)	Firmer fruit	≤	Honeycrisp - SD	Selection mean - SD	10%	25%	25%
M2 (lbs)	Firmer fruit	≤	Honeycrisp - SD	Selection mean - SD	10%	25%	25%
Cn	Various			Keep all			
SSC (°Brix)	High sugar	≤	Honeycrisp - SD	Selection mean - SD	10%	10%	25%
TA (g/L)	High acid	≤	Honeycrisp - SD	Selection mean - SD	10%	10%	25%

a Direction of selection. ≥ indicates that all individuals with observed trait BLUPs or predicted trait values greater than or equal to the threshold value would be designated as culls. ≤ indicates that individuals with observed trait BLUPs or predicted trait values less than or equal to the threshold value would be designated as culls.

b ‘Honeycrisp’s trait BLUPs (Honeycrisp); trait standard deviation (SD); trait BLUP means of advanced selections (Selection mean); percentages refer to percentiles of trait BLUPs of unselected offspring in the training set.

### Traits to target for selection

One of the first challenges is which fruit quality traits to target. Apple fruit quality is affected by a multitude of traits and factors (reviewed by Musacchi and Serra [34]). Additionally, consumers vary in their preferences for fruit with preferences being driven primarily by interactions between texture and taste [33]. Due to variation in consumer preferences, challenges in phenotyping, and incomplete understanding of how traits influence overall eating quality, apple breeders do not always have clear breeding goals or constant weightings for individual traits. Traits that have clear breeding goals (i.e. increase or decrease) like SSC or to an extent flesh firmness (M1, M2), could effectively be targeted. Fruit weight predictions could be used to avoid individuals with the potential to produce very small or excessively large fruits. Although crispness is important, it is a complex trait that is challenging to phenotype. The crispness measure Cn, which was reported to be significantly correlated with sensory crispness (*r* = 0.43 – 0.72) by Evans et al. [35] and Teh et al. [36], had relatively low predictive abilities in this study (Figure 3). In a 2018 study, Chang et al. [37] concluded that no single instrumental measure completely captured sensory crispness in a `Honeycrisp'-derived breeding family. Therefore, multiple instrumental measurements are likely needed to quantify crispness or sensory evaluation should be used. Genomewide prediction of other instrumental measures of crispness and/or sensory crispness should be explored.

## Materials and methods

### Breeding germplasm

Germplasm targeted in this study included 852 unselected offspring from 13 full-sib families representing 14 breeding parents (Table 2). Full-sib family sizes ranged from 23 to 150 unselected offspring with a mean family size of 65 offspring (Table 2). Breeding parents `Honeycrisp' and `Minneiska' (SweeTango® apple), which is an offspring of `Honeycrisp', were highly represented with 528 direct offspring of `Honeycrisp' and 301 direct offspring of `Minneiska' (Table 2). Cultivars AA44 (MonArk), Dayton, Jonafree, Minnewashta (Zestar!® apple), MN55 (First Kiss® or Rave® apple), Pitmaston Pine Apple, WA2 (Sunrise Magic® apple), and Wildung (SnowSweet® apple) and UMN advanced selections MN1702, MN1836, MN1915, and MN1965 were the other parents represented (Table 2). An additional 103 cultivars, progenitors, and advanced selections were included. Individuals were grown on dwarfing rootstocks, typically B.9 or G.16, at the UMN Horticultural Research Center, Chanhassen, MN, USA. All individuals were evaluated at harvest. Kostick and Luby [38] previously analyzed a large subset of this germplasm set to detect and characterize QTLs associated with fruit weight in apple.

### SNP data

As described by Kostick and Luby [38], all individuals included in this study were previously genotyped via the International RosBREED SNP Consortium 8 K Illumina Infinium® array v1 [39] or the Illumina Infinium® 20 K array [40]. The software SNPQC was utilized to impute missing SNP data and remove SNP markers that were monomorphic, had more than 20% missing data, or were redundant with other SNP markers [41]. SNPQC employed a backwards elimination procedure to eliminate redundant SNPs. The SNP with the lower minor allele frequency was eliminated when the correlation between two SNPs was greater than 0.80. The procedure was repeated until the correlations among SNPs were all less than 0.80 and repeated for each of apple’s 17 chromosomes. Unless otherwise noted, 977 SNPs (a mean of 57 SNPs per chromosome) distributed across the 17 apple chromosomes were used in genomewide prediction and postdiction analyses (Table S9). The mean map distance between SNPs was 1.26 cM (Table S9).

### Phenotypic data and analysis

Fruit were evaluated at harvest using the standardized phenotyping protocol described by Evans et al. [42]. Starch iodine index in combination with background color were used to estimate maturity. At least three fruit per individual were harvested at an average starch index rating of approximately four to six on an eight-point scale [43]. Fruit quality traits examined in this study were percent red overcolor, fruit weight, three instrumental fruit flesh texture traits, and two juice chemistry traits at harvest. We focused on fruit quality traits at harvest because at the unreplicated seedling stage of the breeding program, culling or selection decisions are made in the orchard at harvest.

#### Appearance and fruit size traits

Individual fruit were rated for the percentage of the fruit’s surface with red color. Percent red overcolor was rated on a 1 (none) to 5 (≥ 75% red) scale previously described by Evans et al. [42]. Fruit size was estimated from fruit weight as described by Kostick and Luby [38]. Individual fruit were weighed at harvest.

#### Fruit texture traits

Texture analysis was performed using a computerized penetrometer, the Mohr® Digi-Test MDT-1 or, in later years, the MDT-2 (Mohr and Associates, Richland, WA), which have previously been described by Mohr and Mohr [44], Evans et al. [35], and Teh et al. [36] and are briefly summarized here. Teh et al. [36] demonstrated that there was no statistical difference between outputs of MDT-1 and MDT-2. The computerized penetrometer measurements were based on the force encountered by the probe that extends through two of three fruit regions with the skin removed [44]. Region 1 (R1) extends from the outermost region of the fruit to a fixed depth of 0.81 cm (0.32 inches) [44]. Region 2 (R2), which represents most of the edible flesh, extends from R1 to a depth of 30% of the fruit diameter [44]. Similar to Teh et al. [45], the relevant outputs examined in this study were maximum hardness at R1 (M1), maximum hardness at R2 (M2), and a calculated instrumental, unitless crispness value (Cn) [44].

#### Fruit chemistry traits

Evaluation of fruit juice samples for SSC and TA was previously described in detail by Miller et al. [5] and is summarized here. Fruit collected from individuals were pooled and juiced. SSC was estimated by placing approximately 0.2 mL of juice on a handheld refractometer (Atago Digital Hand-held PAL-1). TA was estimated using an automatic titrator (Mettler Toledo T50 and InMotion Flex Autosampler) by titrating 5 mL juice samples with 0.1 M NaOH until the juice sample’s pH reached approximately 8.2. Two technical replicates per sample were evaluated.

#### Statistical analysis of trait data

Prior to statistical analysis, data for percent red overcolor, fruit weight, M1, M2, Cn, SSC, and TA for fruits within a given year were averaged for each individual. Most statistical analyses in this study were carried out in R version 4.0.4 (https://www.r-project.org/). Linear mixed models fit with restricted maximum likelihood (REML) via “lme4” R package [46] were used to analyze mean trait values across years. Year and individual were included as random effects. Models that included year × individual interactions were also initially tested and year × individual interactions were included if inclusion significantly improved model fit (i.e., resulted in a significant decrease in the Akaike Information Criterion value). Normal QQ plots (i.e., sample vs. theoretical quantiles plots) were used to examine normality of random effects (Figure S1). Individual responses were estimated using across year trait best linear unbiased predictions (BLUPs), adjusted by trait means (as described by Amyotte et al. [3], Kostick et al. [47], Kostick and Luby [38]). Adjusted trait BLUPs (i.e., trait BLUPs) of individuals were used as phenotypic values in genomewide prediction and postdiction analyses. Proportions of variance and distributions of phenotypic values were plotted via the R package “ggplot2” [48] and were rendered in Inkscape 1.2 (https://inkscape.org/; accessed 30 January 2023).

### Genomewide prediction – All SNPs included as random effects

Genomewide prediction analyses were conducted within the “rrBLUP” R package [49]. As described by Endelman [49], the mixed.solve() function was applied to calculate solutions for mixed models of the form *y = X*β *+ Zu + ε* where β corresponded to a vector of fixed effects, *u* corresponded to a vector of random effects, and *ε* corresponded to the residual error. In this study, model inputs were *y* which corresponded to the phenotypic trait values (i.e. trait BLUPs) and *Z* which corresponded to the SNP marker matrix of the training set where marker genotypes were coded as 1 and −1 for the homozygous genotypes (i.e. AA, BB) and 0 for heterozygous genotype (i.e. AB). Multiple training – test set scenarios were examined. The Pearson correlation coefficient (*r*) between the predicted and observed (trait BLUPs) trait values for individuals in the test set was used to calculate model predictive ability.

#### All families

Genomewide prediction models were tested using all unselected offspring via two training-test set schemes: 1) random samples of 10, 25, 50, or 75% of population as training sets and 2) all but one untested family as the training set. In the random subset method, a proportion of the population was randomly selected to serve as the training set while the remaining proportion of the population was used as the test set. For example, at the 10% sampling level, a random sample of 10% of the population was used as the training set while the remaining 90% were used as the test set. For each sampling level (i.e. 10, 25, 50, 75%), 500 iterations were completed. In the untested family method, one family served as the test set while the remaining families were used to train the model. For example, if the `Honeycrisp' × `Minnewashta' family was considered the test set, offspring from all remaining families would be used as the training set. This procedure was repeated so that each family served as the test set.

#### Within families

Utility of genomewide prediction models for prediction of fruit quality traits within families was tested by a delete-one procedure. For example, when testing a genomewide prediction model within the `Honeycrisp' × `Minnewashta' family (n = 150), the performance of each offspring was predicted from the performance of the remaining 149 offspring.

To examine the effect of family size on predictive ability of within-family genomewide prediction, random samples of 20, 40, 60, 80, 100, and, if possible, 120 offspring from large full-sib families (i.e., `Honeycrisp' × `Minnewashta', `Minneiska' × `MN55') were selected to represent the family. For each sampling level, 100 iterations were completed. Genomewide prediction models were tested within these subsets using single untested offspring from the subset as the test set and the remaining offspring as the training set. This procedure was repeated until all offspring had been used as the test set.

#### Number of SNPs per chromosome

To examine the effect of number of SNPs per chromosome on model predictive ability, random samples of five, 10, 15, 20, 25, and 30 SNPs per chromosome were included as random effects in the genomewide prediction model. SNPs were sampled from the 977 SNPs retained after curation with SNPQC software. One family served as the test set while the remaining families were used to train the model. For each sampling level, test set, and trait combination, 100 iterations were completed.

### Genomewide prediction – SNPs at large-effect QTLs included as fixed effects

For percent red overcolor, TA, M1, and M2, individual SNPs near or within previously reported large-effect QTL regions were selected. Specifically, SNPs near the Chr9 color locus [e.g., 16, 19, 29], Chr8 (*Ma3)* and Chr16 (*Ma*) [e.g., 30, 50, and Chr16 texture locus near *Ma* [51], loci were examined. Analysis of variance (ANOVA) was used to determine if there were significant differences for mean trait values among genotypes at selected SNPs. Tukey’s honestly significant difference (HSD) test applied via the R package “agricolae” [52] was used for mean separation among genotype groups. Selected SNPs were then included as fixed effects in genomewide prediction models. The model.matrix() function in R was used to determine the design matrix for fixed effects. All other SNPs were included as random effects. The mixed.solve() function from the rrBLUP package [49] was applied to calculate solutions of genomewide prediction models with fixed effects. Model inputs were *X* which corresponded to the design matrix for the fixed effect SNP(s) and previously described *y* and *Z*. One family served as the test set while the remaining families were used to train the model. This was repeated until all families had been used at the test set. Paired *t-tests* were used to determine if there were significant differences in model predictive abilities between models with SNPs at large-effect QTLs included as fixed effects versus models that included all SNPs as random effects.

### Genomewide postdiction

To examine the utility of genomewide prediction within the context of an apple breeding program, postdiction (retrospective) analyses were carried out to determine if advanced selections identified via traditional selection procedures would have been kept or culled via genomewide selection for a set of fruit quality traits.

#### Training and test sets

In addition to the 852 unselected offspring from the 13 full-sib families (Table 2), 92 advanced selections, cultivars, progenitors, and parents were included in postdiction analyses. Advanced selections were grouped into four groups based on the year (i.e. pre-1995, 1995 to 2005, 2006 to 2015, 2016 to 2020) they were advanced from the first (i.e. unreplicated seedling phase) to the second phase (i.e., small, replicated plantings) of evaluation in the breeding program. Only advanced selections with data for most target traits (i.e. percent red overcolor, fruit weight, M1, M2, Cn, SSC, TA) were included in postdiction analyses. Three sets of individuals in training and test sets were considered in postdiction analyses. In the first set, unselected offspring from nine full-sib families (Table 2), 12 cultivars, progenitors, or parents, and 11 individuals advanced as selections prior to 1995 were included in the training set while 16 advanced selections selected from 1995 to 2005 were included as the test set. In the second set of postdiction analyses, the training set consisted of the training and test sets from set one plus offspring from `Minneiska'-derived full-sib families while 47 advanced selections selected from 2005 to 2016 were included as the test set. For the final set of postdiction analyses, the training set consisted of the training and test sets from round two and the test set was six advanced selections selected from 2016 to 2020.

#### Comparison of phenotypic and genomewide selection

Fruit weight, M1, M2, SSC, and TA were selected because they had breeding relevance, sufficient phenotypic variation, moderate to high observed predictive abilities, or lacked available relevant locus-specific DNA tests. Five different culling threshold scenarios relevant to the breeding program were examined in this study and are described in Table 3. For each culling threshold scenario, independent culling thresholds were estimated for each trait. The numbers of unselected offspring or advanced selections that would have been culled or kept based on observed or predicted trait values were determined for each trait separately and across all targeted traits. The total numbers of individuals that would have been culled and kept based on phenotypic and genomewide selection procedures were compared. All genomewide prediction and postdiction figures were plotted via R package “ggplot2” [48] and rendered in Inkscape 1.2 (https://inkscape.org/; accessed 30 January 2023).

## Supplementary Material

Web_Material_uhad088

## Data Availability

The data presented in this study are available in the tables, figures, and supplementary materials.
