# Supplementary material for: Genomewide selection for fruit quality traits in apple: breeding insights gained from prediction and postdiction
Source: Hortic Res. 2023 May 3;10(6):uhad088. doi: 10.1093/hr/uhad088 (PMC10273070; doi:10.1093/hr/uhad088)

Figure S1. Normal QQ plots of random effects in linear mixed models for percent red overcolor (S1.1), fruit weight (S1.2), firmness measure M1 (S1.3), firmness measure M2 (S1.4), instrumental crispness estimate Cn (S1.5), soluble solids content (S1.6), and titratable acidity (S1.7) trait data. QQ plots were used to check normality of random effects.

S1.1

Percent red overcolor

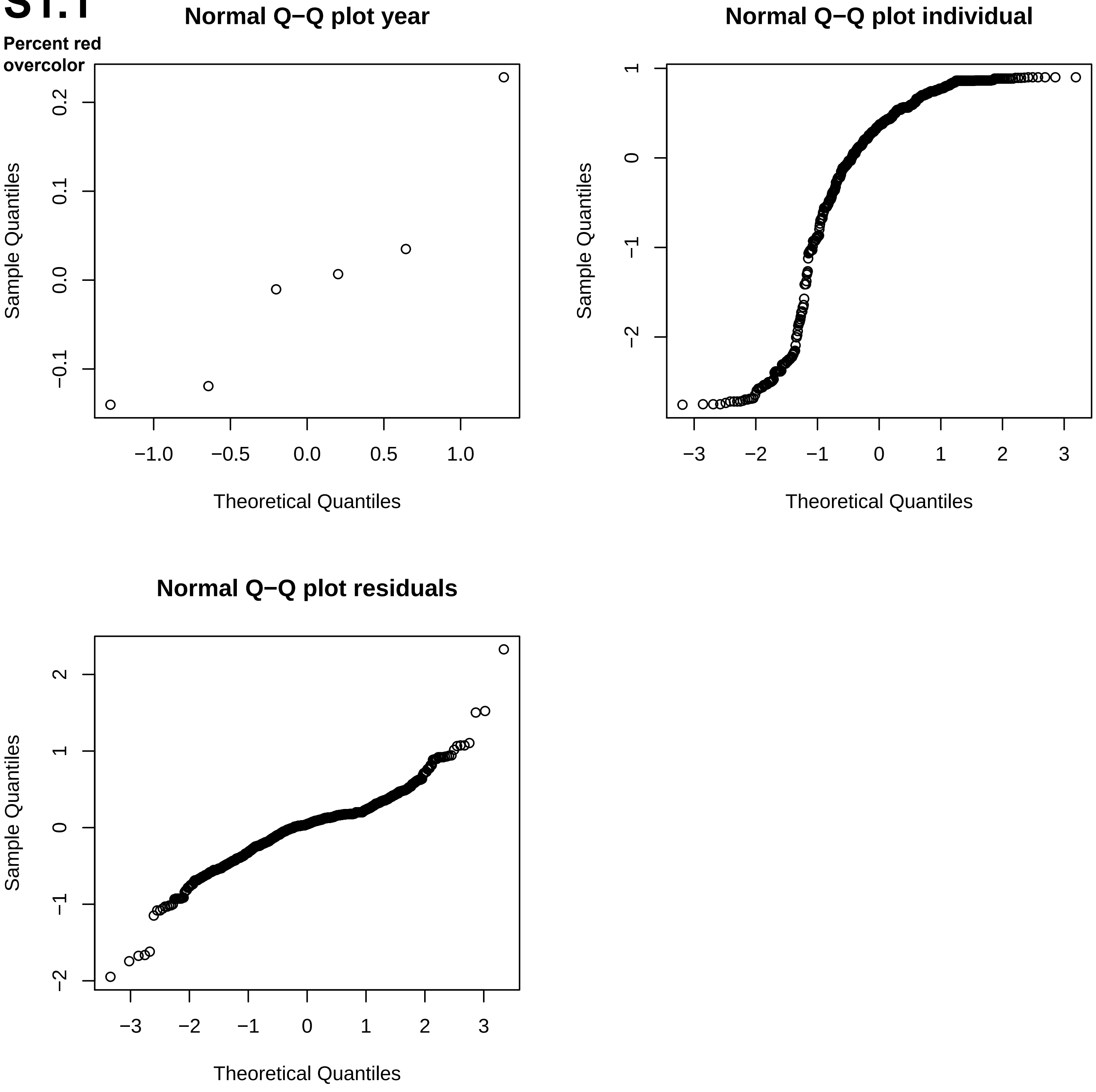

S1.2

Fruit weight

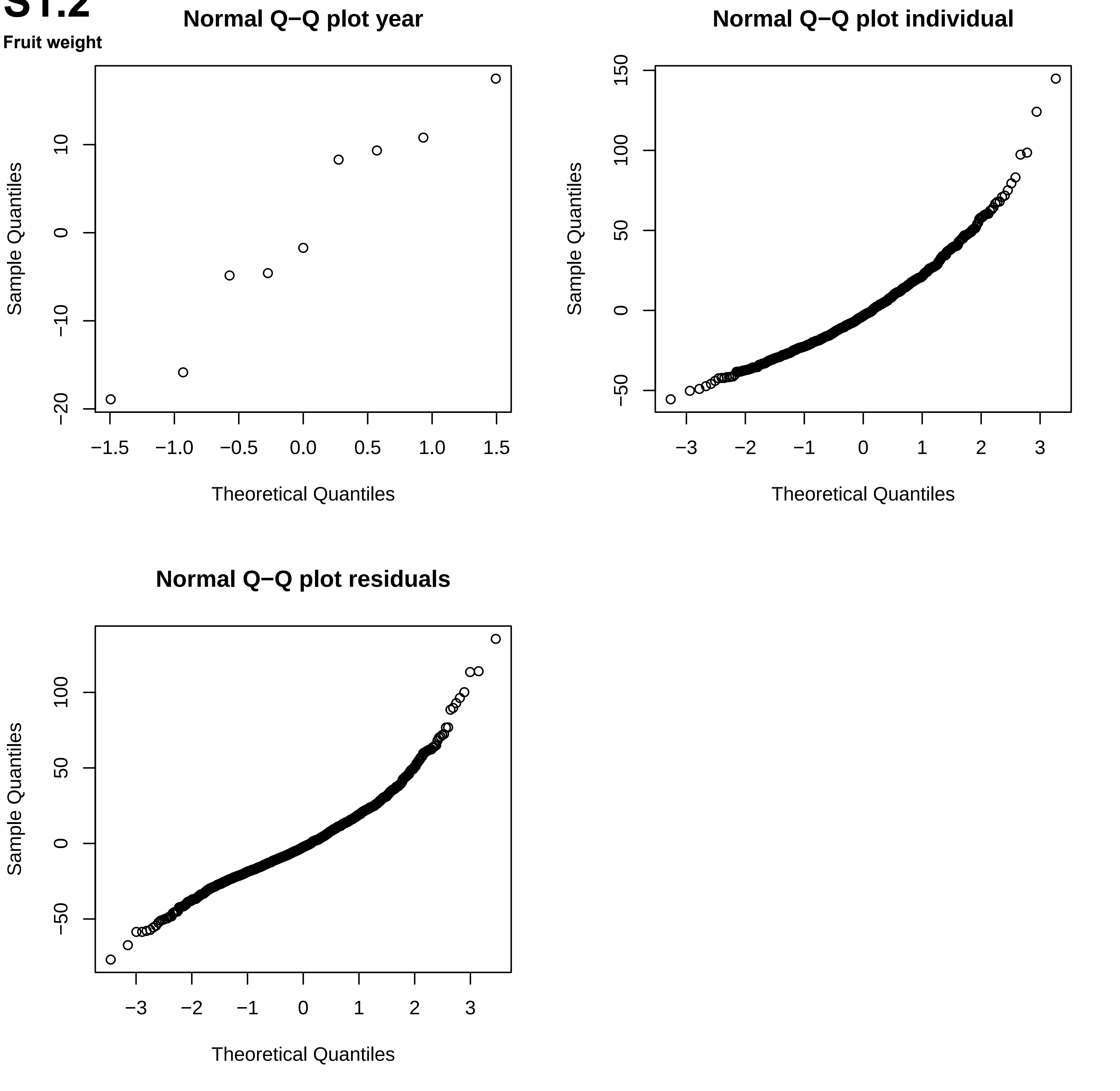

S1.3

Firmness M1

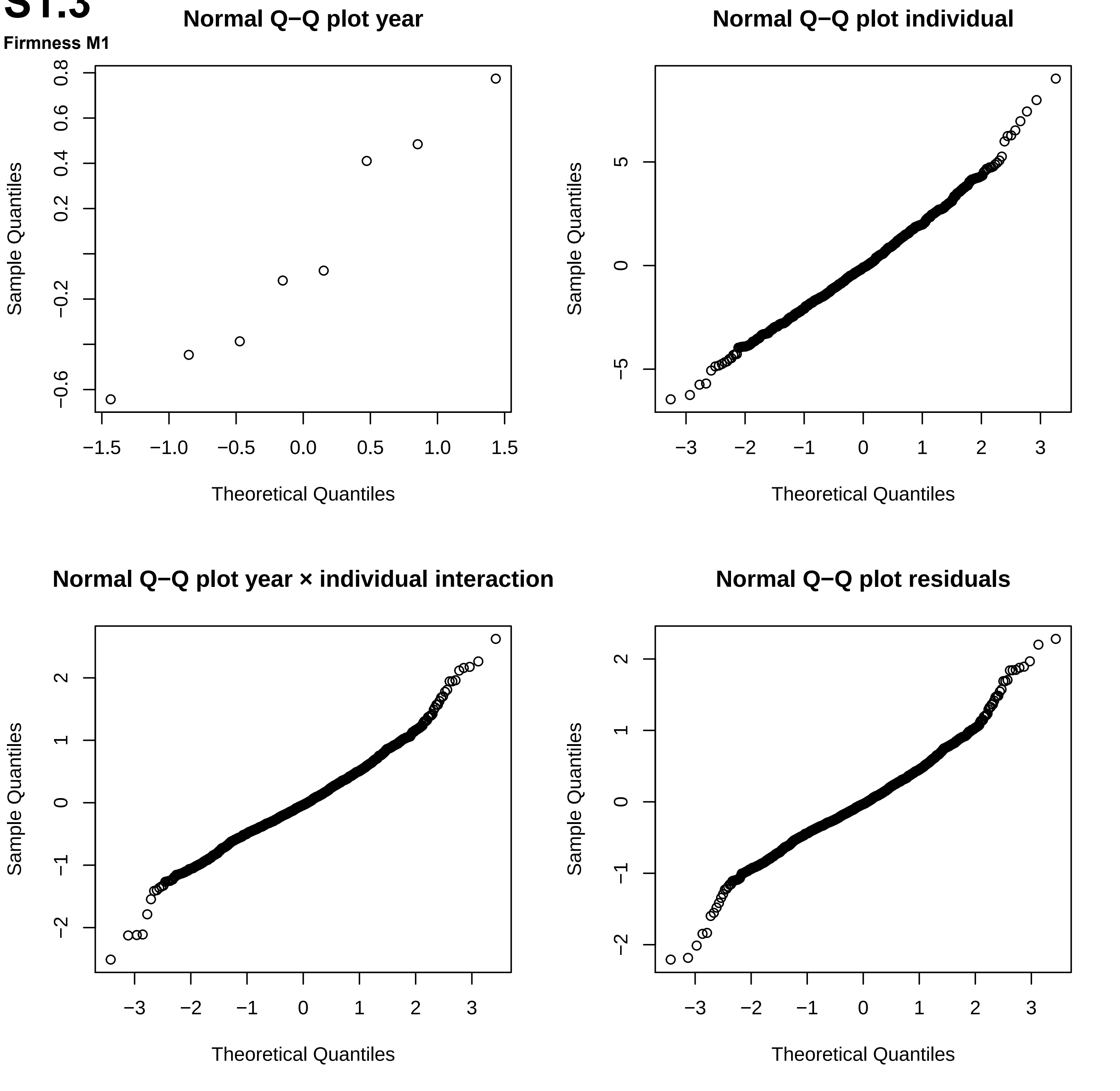

S1.4

Firmness M2

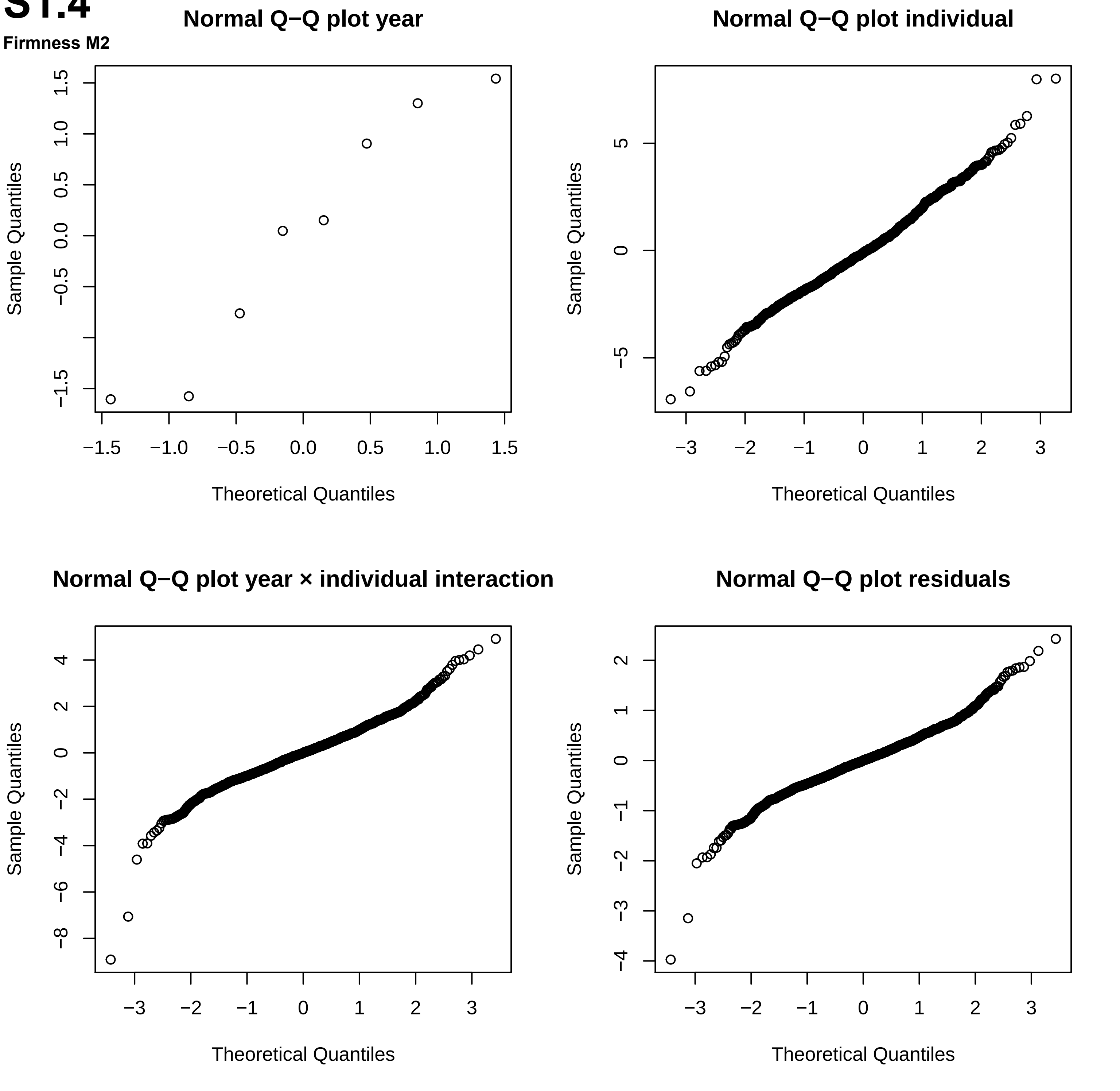

S1.5

Crispness Cn

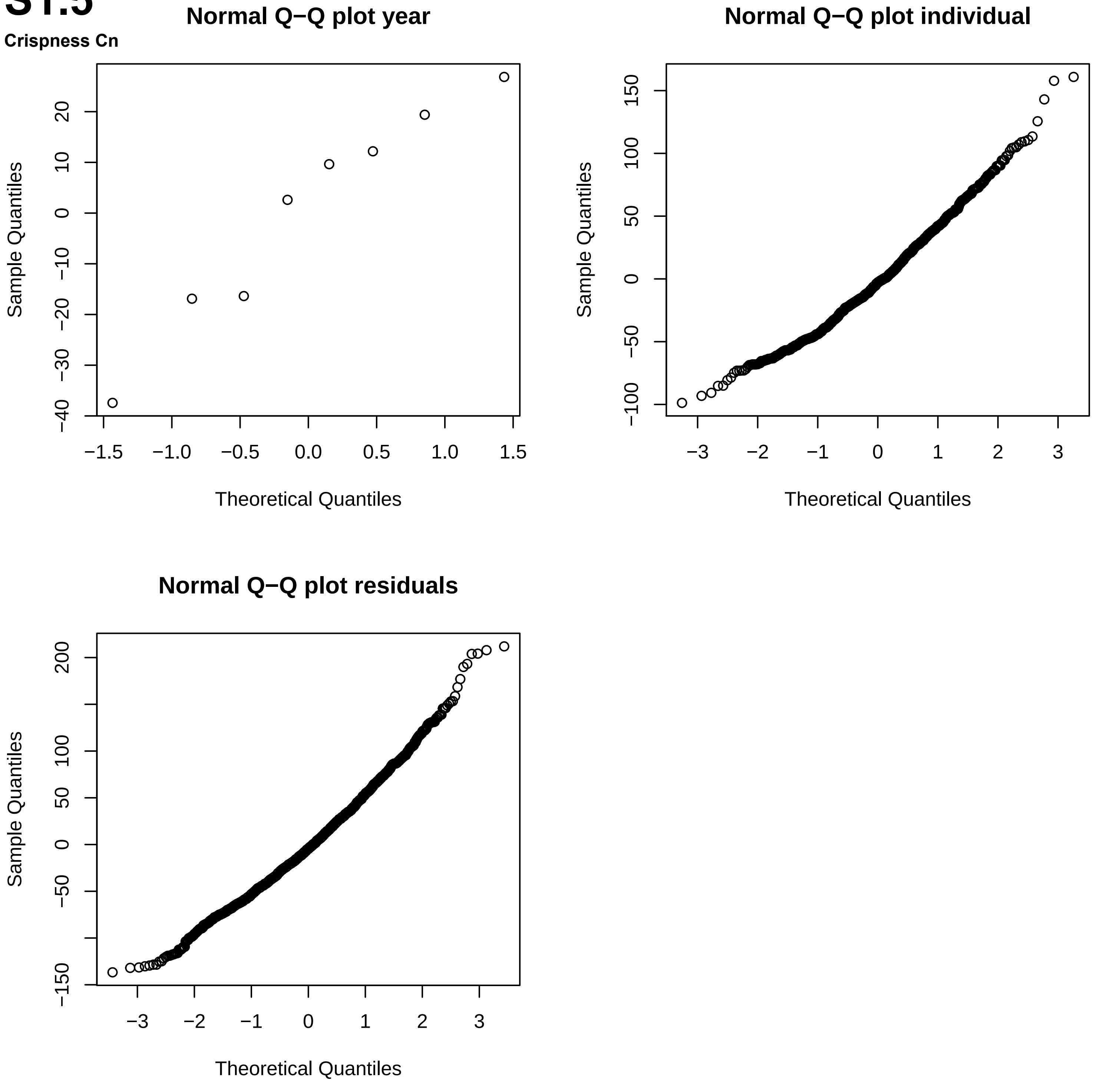

S1.6

SSC

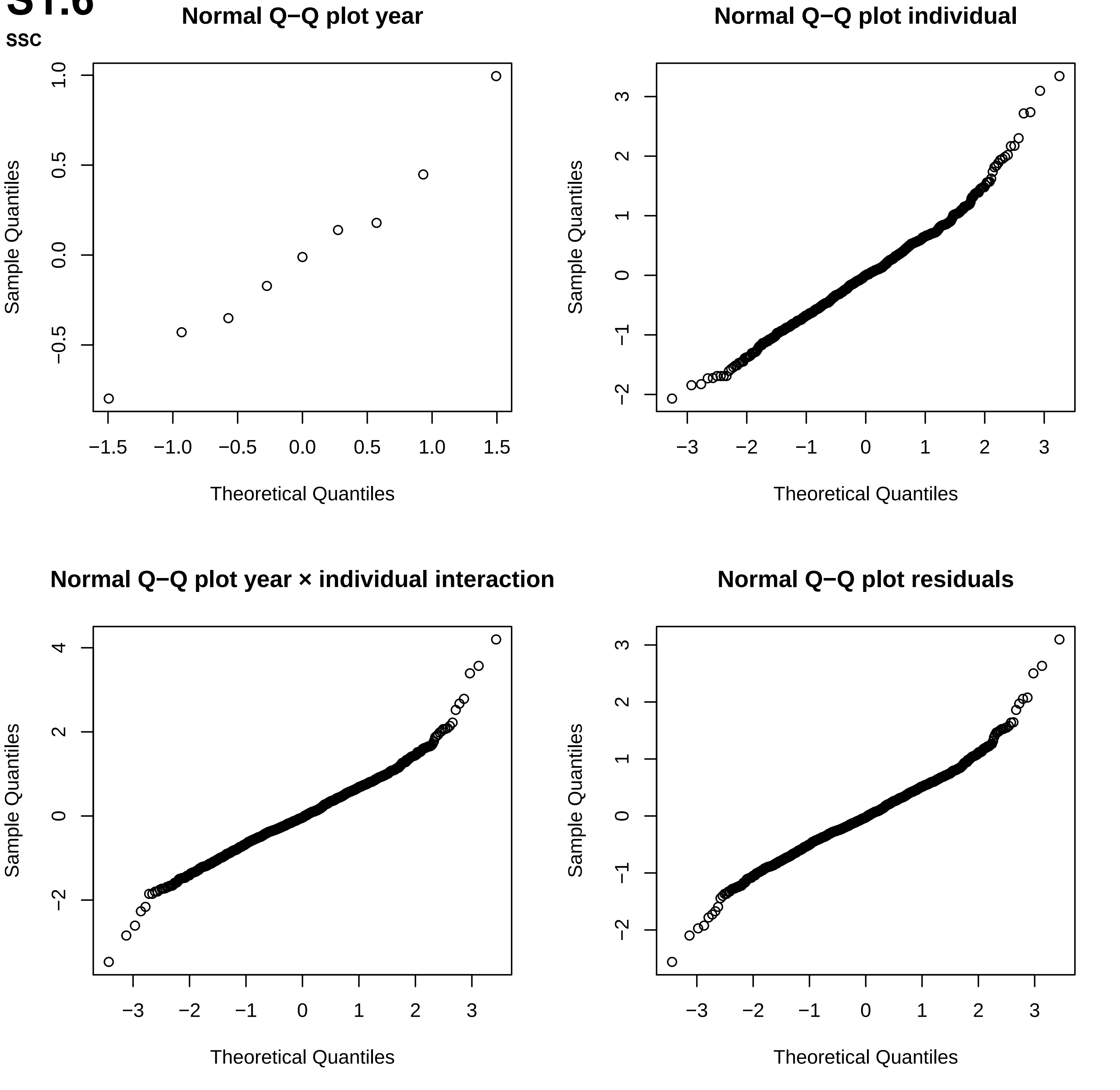
Normal Q−Q plot year
Normal Q−Q plot individual
Normal Q−Q plot year × individual interaction
Normal Q−Q plot residuals
Sample Quantiles
Theoretical Quantiles
−1.5
−1.0
−0.5
0.0
0.5
1.0
1.5
−3
−2
−1
0
1
2
3
4

S1.7

TA

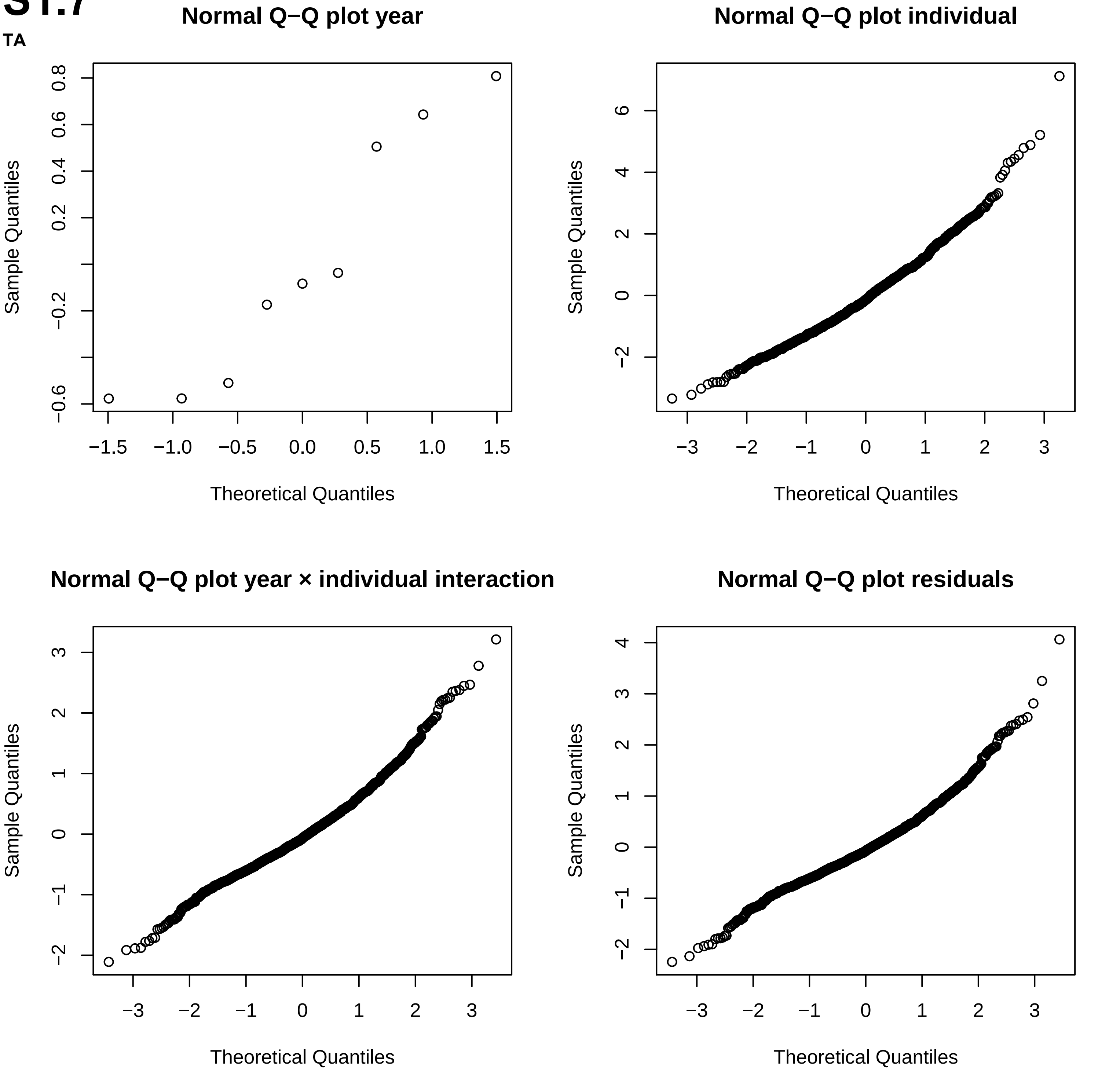
Normal Q−Q plot year
Normal Q−Q plot individual
Normal Q−Q plot year × individual interaction
Normal Q−Q plot residuals
Sample Quantiles
Theoretical Quantiles
−0.6
−0.2
0.2
0.4
0.6
0.8
−1.5
−1.0
−0.5
0.0
0.5
1.0
1.5
−3
−2
−1
0
1
2
3
4
6

Figure S2. Effects of SNP number per chromosome on genomewide prediction model predictive abilities for fruit quality traits at harvest. Random samples of SNPs per chromosome were included as random effects in genomewide prediction models. N-1 families served as the training set while the remaining family served as the testing set. This was repeated so that each family served as the testing set. For each sampling level, test family, and trait combination, 100 iterations were completed. The red solid line is the mean predictive ability for a given trait across test families when all SNPs were included in genomewide prediction models. The red dashed line is the mean predictive ability minus the standard deviation for a given trait across test families when all SNPs were included in genomewide prediction models.

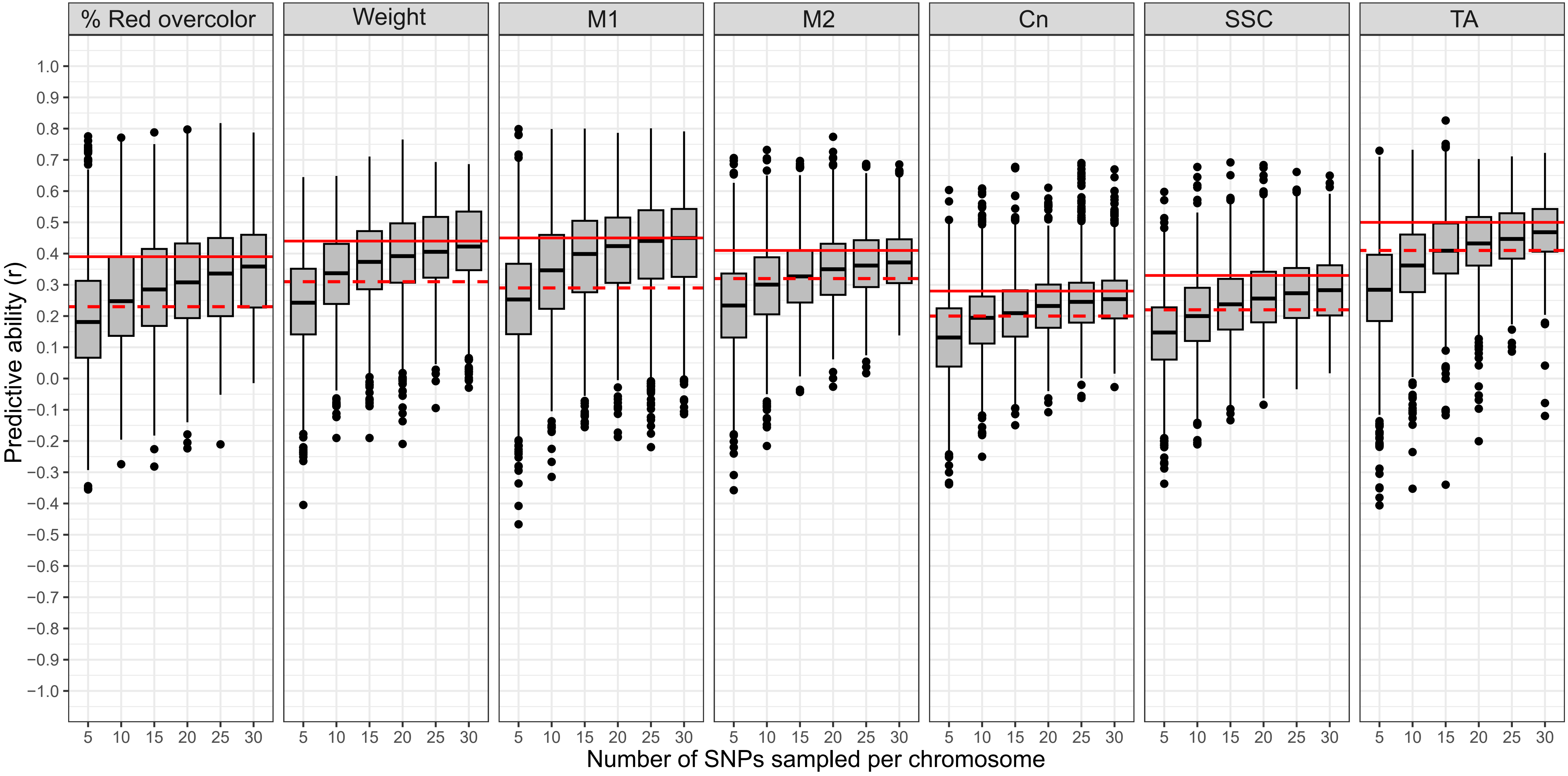

Figure S3. Effect of population size on within family genomewide prediction model predictive abilities for fruit quality traits at harvest. Random samples of offspring from 'Honeycrisp' × 'Minnewashta' full-sib family were selected to represent family. For each sampling level, 100 iterations were completed. Genomewide prediction models were tested within these subsets by using single untested offspring as the test set and the remaining offspring as the training set. This was repeated until all offspring in the subset had been used as the testing set. The red dashed line is 90% of within family predictive ability when all but one offspring (N-1 offspring) were used to train the within family genomewide prediction models.

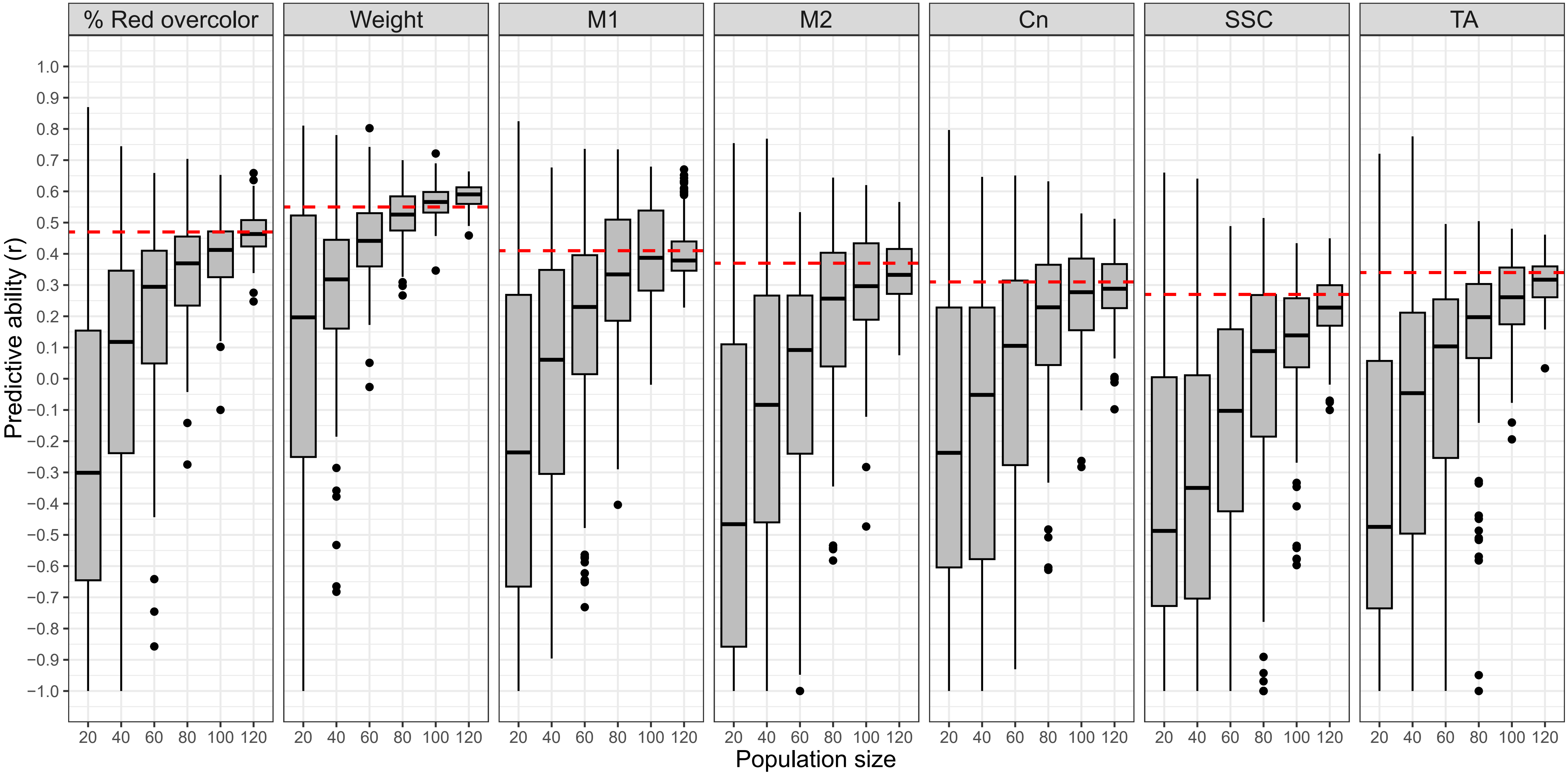

Figure S4. Effect of population size on within family genomewide prediction model predictive abilities for fruit quality traits at harvest. Random samples of offspring from 'Minneiska' × 'MN55' full-sib family were selected to represent family. For each sampling level, 100 iterations were completed. Genomewide prediction models were tested within these subsets by using single untested offspring as the test set and the remaining offspring as the training set. This was repeated until all offspring in the subset had been used as the testing set. The red dashed line is 90% of within family predictive ability when all but one offspring (N-1 offspring) were used to train the within family genomewide prediction models.

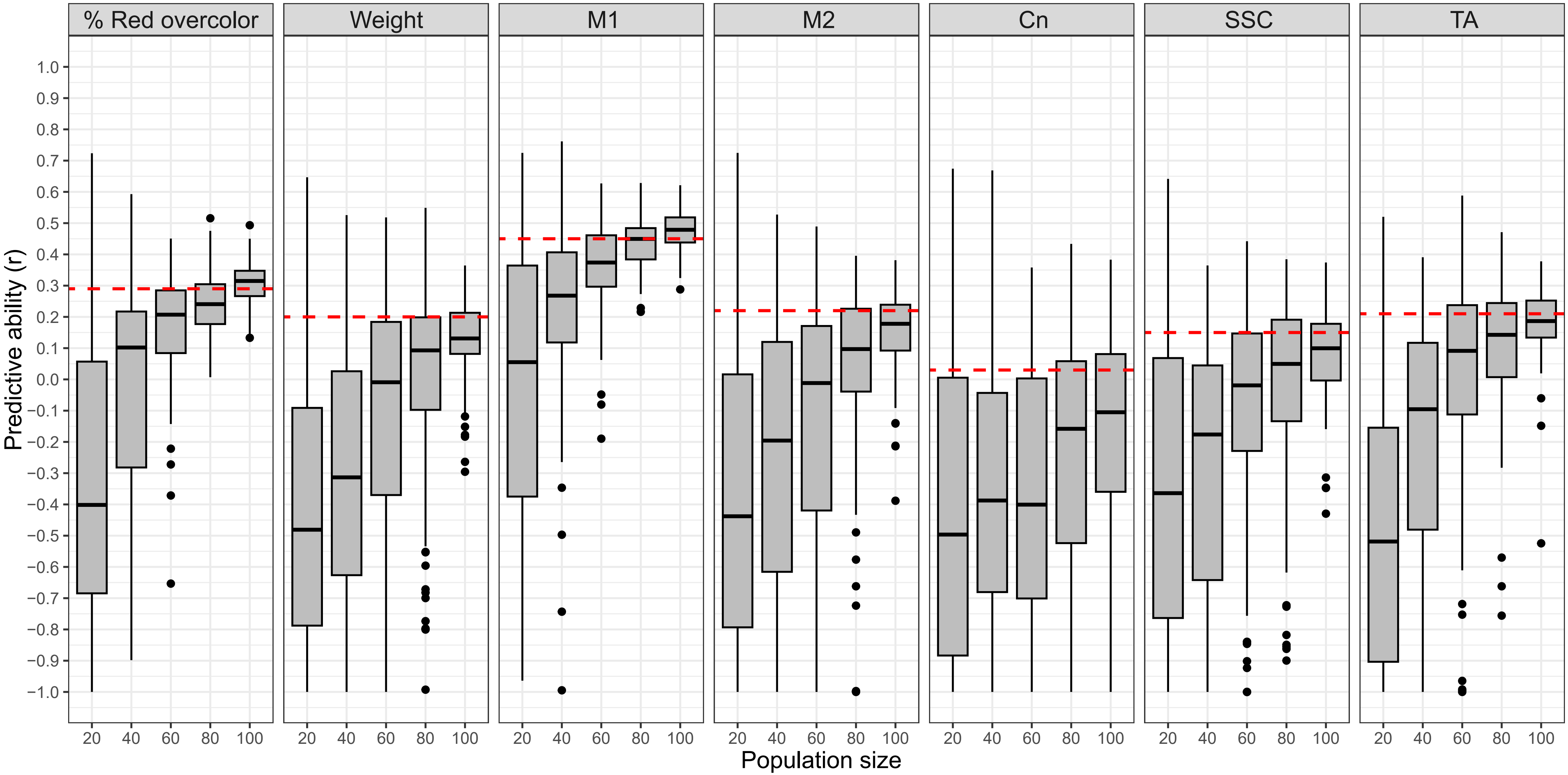

Supplement: Web_Material_uhad088 [file web_material_uhad088.zip › Figures S1-S4 combined.pdf]
